# The Advocacy for Pedestrian Safety Study: Cluster Randomised Trial Evaluating a Political Advocacy Approach to Reduce Pedestrian Injuries in Deprived Communities

**DOI:** 10.1371/journal.pone.0060158

**Published:** 2013-04-08

**Authors:** Ronan A. Lyons, Denise Kendrick, Elizabeth M. L. Towner, Carol Coupland, Mike Hayes, Nicola Christie, Judith Sleney, Sarah Jones, Richard Kimberlee, Sarah E. Rodgers, Samantha Turner, Mariana Brussoni, Yana Vinogradova, Tinnu Sarvotham, Steven Macey

**Affiliations:** 1 Swansea University, Swansea, Wales, England; 2 Nottingham University, Nottingham, England; 3 University of West of England, Bristol, England; 4 Child Accident Prevention Trust, London, England; 5 University College London, London, England; 6 University of Surrey, Surrey, England; 7 Cardiff University, Cardiff, Wales, England; 8 University of British Columbia, Vancouver, Canada; Cardiff University, United Kingdom

## Abstract

**Objective:**

To determine whether advocacy targeted at local politicians leads to action to reduce the risk of pedestrian injury in deprived areas.

**Design:**

Cluster randomised controlled trial.

**Setting:**

239 electoral wards in 57 local authorities in England and Wales.

**Participants:**

617 elected local politicians.

**Interventions:**

Intervention group politicians were provided with tailored information packs, including maps of casualty sites, numbers injured and a synopsis of effective interventions.

**Main outcome measures:**

25–30 months post intervention, primary outcomes included: electoral ward level: percentage of road traffic calmed; proportion with new interventions; school level: percentage with 20 mph zones, Safe Routes to School, pedestrian training or road safety education; politician level: percentage lobbying for safety measures. Secondary outcomes included politicians’ interest and involvement in injury prevention, and facilitators and barriers to implementation.

**Results:**

Primary outcomes did not significantly differ: % difference in traffic calming (0.07, 95%CI: −0.07 to 0.20); proportion of schools with 20 mph zones (RR 1.47, 95%CI: 0.93 to 2.32), Safe Routes to School (RR 1.34, 95%CI: 0.83 to 2.17), pedestrian training (RR 1.23, 95%CI: 0.95 to 1.61) or other safety education (RR 1.16, 95%CI: 0.97 to 1.39). Intervention group politicians reported greater interest in child injury prevention (RR 1.09, 95%CI 1.03 to 1.16), belief in potential to help prevent injuries (RR 1.36, 95%CI 1.16 to 1.61), particularly pedestrian safety (RR 1.55, 95%CI 1.19 to 2.03). 63% of intervention politicians reported supporting new pedestrian safety schemes. The majority found the advocacy information surprising, interesting, effectively presented, and could identify suitable local interventions.

**Conclusions:**

This study demonstrates the feasibility of an innovative approach to translational public health by targeting local politicians in a randomised controlled trial. The intervention package was positively viewed and raised interest but changes in interventions were not statistically significance. Longer term supported advocacy may be needed.

**Trial Registration:**

Current Controlled Trials ISRCTN91381117

## Introduction

The importance of translating public health research to maximise the health benefits of effective interventions is increasingly being recognised.[1]–[2] Road traffic injury is a major global public health problem and a leading cause of death amongst children and young people.[3]–[5] Road traffic related injuries, particularly for child pedestrians, are among the greatest of all health inequalities, with much higher rates in children from families led by parents in unskilled employment or from deprived neighbourhoods.[3], [6]–[8], [4], [9]–[10] There are a range of effective interventions available yet their implementation is often suboptimal or is not appropriately targeted towards deprived areas with the highest pedestrian casualty rates.[11]–[19].

To date, little research has been undertaken on translational public health approaches assessing methods for increasing uptake of effective interventions in high risk communities. [20] In the context of road safety, a longitudinal ecological UK study found that traffic calming in disadvantaged communities was associated with reductions in absolute child pedestrian injury rates and relative inequalities. [21] Secondary analysis showed higher rates of traffic calming in areas represented by influential local politicians suggesting that political advocacy may be effective in implementation of road safety measures. [22] This would be consistent with individual-level behaviour-change models which suggest that an advocacy approach engaging local politicians should encourage action to improve safety, if they were provided with information that the areas they represent had particularly high injury rates and the means to improve safety was within their sphere of influence. [23].

Advocacy emerged as a public health promotion strategy in the 1980s.[24]–[25] Carlisle considers that the role of health advocacy is: “to influence governments and national/international agencies in beneficent and health-promoting ways, and to raise the profile of health-promoting organizations, ensuring that their voices are heard and taken note of.” [26] Advocacy’s important role in injury prevention is widely recognised, but there are few rigorous studies of advocacy published in this field.[27]–[30] The only randomised trial evaluating political advocacy that we could find sent briefing letters to Illinois senators in 1982 which led to increased support for legislation on child safety restraints. [31].

The Advocacy for Pedestrian Safety Study adopted political advocacy as a promising approach to implement translational public health research in an attempt to improve pedestrian safety in high risk communities in the UK. We developed a package to promote advocacy for effective pedestrian safety interventions and undertook a cluster randomised controlled trial to assess the effectiveness of this approach in improving pedestrian safety in disadvantaged communities. The intervention was directed at local politicians who represented electoral wards and worked within local authorities. In the UK, decisions on road safety strategy and implementation of interventions are taken at local authority level; hence the local authority was the unit of randomisation.

The objectives of the trial were:

1. To identify areas (electoral wards) represented by local politicians in deprived communities with a history of high pedestrian injury rates among vulnerable road users.
2. To develop a package to promote advocacy for implementation of effective pedestrian safety interventions by local politicians.
3. To undertake a cluster randomised controlled trial to test the efficacy of the advocacy package
4. To explore factors related to the success or failure of the intervention.

## Methods

The protocol for this trial and supporting CONSORT checklist are available as supporting information; see Checklist S1 and Protocol S1.

### Design

A detailed methodology for this study has been published. [32] The ‘Advocacy for Pedestrian Safety Study’ was designed as a multi-centre mixed methods study incorporating a cluster randomised controlled trial. The study took place in 4 centres: South Wales, and areas of the South West, East Midlands, and South East of England, within 50km of the universities of Swansea, Cardiff, the West of England-Bristol, Nottingham and Surrey.

### Participants

Participants were elected local politicians representing deprived electoral wards which had high pedestrian injury rates in 2000–2003 for vulnerable groups (children aged 4–16 years and adults over 60s) in local authorities in the four areas of the UK described above. There are different local government arrangements within England and between England and Wales. Multi-tier local authorities are common in parts of England whereas single tier authorities operate throughout Wales and parts of England. Multi-tiered authorities involve a complex mixture of responsibilities divided between counties (higher tier) and districts (lower tier). Road safety is usually the responsibility of the higher tier but is often shared between tiers. Local politicians are elected to represent electoral wards in both tiers of government. County wards are generally larger than district wards. A county ward may overlap with two or more district wards. Local politicians are elected to represent district or county wards, and in some cases represent both. All local politicians representing electoral wards at district level or county wards which covered all or part of the district electoral wards in the study areas were included. The district local authority was chosen as the unit of randomisation as this was common across all areas.

Vulnerable pedestrian casualty rates (aged 4 to 16 years and 60+ years ) were calculated using police recorded road crash statistics (STATS19) for 2000–2003, held in the UK data archive (UK data archive). [33] Data for pedestrian casualties were mapped onto the boundaries of the 8800 electoral wards in England and Wales using ArcView 3.2. Each casualty was assigned to an electoral ward, casualties per electoral ward aggregated and rates per 1000 population calculated using population estimates from the 2001 census.

Deprivation scores in the form of Townsend Index Scores were obtained for each of the 8800 wards. [34] The Townsend Index was devised by Townsend et al in 1988 to provide a material measure of deprivation and disadvantage. The Index is based on four different variables taken, originally from the 1991 UK Census. [34]. The four variables that comprise the Townsend Index are: unemployment as a percentage of those aged 16 and over who are economically active; non-car ownership, as a percentage of all households; non-home ownership as a percentage of all households; and household overcrowding. Z scores are used to standardize the component variables. The z score is simply the ‘observation’ (percentage or proportion for the ward on a given measure) minus the mean observation divided by the standard deviation. The Townsend Score is a summary of the four component z scores.

The 8800 wards were then ranked by the deprivation scores and vulnerable casualty rates. Electoral wards in the most deprived third with injury rates in the highest third were then identified (n = 1902) and distance to the nearest study centre calculated.

To facilitate data collection, only electoral wards within 50km of one of the four study centres were eligible for inclusion (n = 319). These were then grouped into local authorities and the numbers of eligible electoral wards within each local authority calculated. Where more than 8 electoral wards in any local authority were eligible, 8 were randomly selected for inclusion to reduce burden on authorities with limited resources and capacity for action.

### Interventions

The intervention group (all local politicians representing intervention electoral wards within intervention local authorities) received a postal package to promote advocacy in October 2005. This contained tailored information, specific to their electoral ward, as well as general pedestrian injury information. Specific information included the high injury rate, a map of vulnerable pedestrian injury locations for their electoral ward for 2000–2003, and the estimated monetary value of preventing such injuries. General information included pedestrian injury risk factors, details of evidence based interventions, the role of local government in implementation and advice on who to contact within the local authority to facilitate action. An example of an intervention information package is included in Appendix S1. Information in the package was reinforced during a telephone interview 1–3 months later. Control group local politicians received general information on children’s home and road injuries and advice on prevention measures and government policy from the Child Accident Prevention Trust, shown in Appendix S2. Control groups did not receive any information specific to their wards.

### Outcomes

Primary outcomes were measured at the electoral ward, school and local politician levels and comprised:

A. Electoral ward level

1. The percentage of kilometres of road that were traffic calmed per ward.
2. A composite outcome measure comprising the proportion of wards where any new road safety interventions were introduced.

B. School level

1. The percentage of schools with 20 mph zones.
2. The percentage of schools with a Safe Routes to School initiative.
3. The percentage of schools providing practical pedestrian training.
4. The percentage of schools providing other road safety education.

C. Local politician level

1. The percentage of local politicians who lobbied for physical road safety measures or more road safety education in their wards.

The number of traffic calming features was specified as a primary outcome measure in the planning phase as these data were available in 2005. However, in 2006 the Ordnance Survey stopped collecting these data; hence the number of kilometres of road with traffic calming features and the total number of kilometres of road per ward were used as these were available for 2005 and 2007. The Ordnance Survey divides all roads into segments which are the road lengths between consecutive junctions. The data contain an indicator as to whether (and when) each segment has been traffic calmed using any type of vertical hump.

Secondary outcomes were measured at school or local politician level and comprised:

A. School level

1. The percentage of schools with 20 mph zones planned.
2. The percentage of schools with a Safe Routes to School initiative planned.
3. The percentage of schools with practical pedestrian training planned.
4. The percentage of schools at follow-up in process of making a school travel plan.
5. The percentage of schools at follow-up planning one or more of the above measures

B. Local politician level.

1. Interest in child injury prevention.
2. Involvement in child injury prevention in the preceding 12 months.
3. Beliefs that they could take action to help prevent child injuries in their electoral wards.
4. Specific mention of pedestrian safety as one action for preventing child injuries in their electoral wards.
5. Identification of barriers and facilitators to initiating and planning pedestrian safety improvement in electoral wards.

Changes in the distribution of traffic calming were assessed 25–30 months post intervention through analysis of UK Ordnance Survey MasterMap data which are updated on a six monthly basis (Ordnance Survey, 2007). [35].

Data on school level outcomes was ascertained from postal survey with telephone follow up of local authority road safety departments 28–30 months post intervention and from a postal survey of head teachers of 757 schools whose catchment areas were likely to include the study electoral wards between 25 and 27 months post intervention. The survey instruments distinguished between interventions which preceded or were put in place during the study. Data on local politician level outcomes was ascertained from semi-structured telephone interviews and a postal survey 1–3 months post provision of the information and advocacy package, and semi-structured telephone interviews 17–22 months post intervention in the intervention group, and a postal survey 25–27 months post intervention in both intervention and control groups. The baseline interviews to all councillors were based on structured questionnaires which sought to explore the relative importance of road safety issues amongst other common issues in neighbourhoods based on the Audit Commission’s quality of life survey and on current provision for child pedestrian a safety such as safe routes to school, pedestrian training and 20 mph zones. [36]. Subsequent questionnaires sought similar information to see how responses to this changed among councillors in the different treatment groups. Among councillors who had received the tailored information about child road safety in their ward semi-structured questionnaire based interviews were used to explore their views about the information pack such as whether or not they had found it interesting, if they had learned anything new, was it presented effectively and what were their plans for child road safety. For these open response questions a coding frame was developed based on initial interviews so that the responses could be categorised into positive and negative aspects and as a means of characterising their views on how they were going to address children’s road safety.

### Methods used to Enhance the Quality of Measurements

All questionnaire and interview schedules and the contents of the package to promote advocacy were pilot tested on local politicians, road safety officers, and teachers from outside the study areas and subsequent modifications made.

### Samples Size and Interim Analyses

The estimated sample size for this study was 117 electoral wards per treatment group. Sample size calculations were based on the results of an earlier pilot study undertaken in two areas of Wales. This pilot study measured the number of traffic calming features in electoral wards and found that there were on average 21 features per electoral wards (SD 27.2). [32] An effect considered to be of public health importance would be a standardised difference of 0.35 between the mean number of new traffic calming features in intervention and control electoral wards. [37] Using this as the measure of effect, then a 1 sided significance test (based on assumption that the intervention can only improve new traffic calming features) at α = 0.05 and power of 80% requires 102 electoral wards in each treatment group. Assuming an average of 4 electoral wards per local authority and an intra class correlation coefficient of 0.05, the design effect is 1.15 and the required sample size is 117 electoral wards per group. [38] No interim analyses were performed.

### Randomisation

Local authorities were randomised to intervention or control groups, stratified by study centre (4 strata) and local authority size (2 strata: 1–3 electoral wards; more than 3 electoral wards). The randomisation schedule was computer generated using the StatsDirect package by a statistician (CC), blind to the identity of the local authorities. Randomisation was blocked within each stratum to ensure equal numbers of local authorities in each arm of the study. The block size was the number of local authorities in the stratum, if even or the number +1 if odd. This ranged from 5 to 14.

### Allocation Concealment

A study team member (DK) generated random numbers for each local authority to allocate them to treatment groups. The randomisation schedule was blinded to the identity of the local authority. This list was then merged with a separate file containing the identity of the local authority.

### Blinding

It was not possible to blind local politicians to treatment group allocation but they were not informed that they were in a comparative study. Teachers and road safety officers were blinded to intervention status. Analyses were undertaken masked to treatment group allocation.

### Statistical Methods

Analyses were undertaken according to a predefined analysis plan. The data on the percentage of kilometres of road traffic calmed were highly skewed and a cube root transformation was used in a random effects linear regression analysis as this satisfied the assumptions of the analysis. The analysis accounted for clustering of wards by local authority, adjusted for the cube root of the percentage of kilometres of road traffic calmed at baseline (2005) and also for randomisation strata as a fixed effect.

As positive responses were common for binary outcomes, relative risks were estimated rather than odds ratios using two-level log-binomial generalised estimating equations. Where there were problems with convergence, Poisson generalised estimating equations with a robust variance estimator were used.[39]–[40] All analyses were adjusted for stratum and for clustering at local authority level. Analyses were repeated assuming those with missing values, had and did not have the outcome of interest. Data were analysed using Stata version 10.

## Results

### Participant Flow

**Figure 1:**
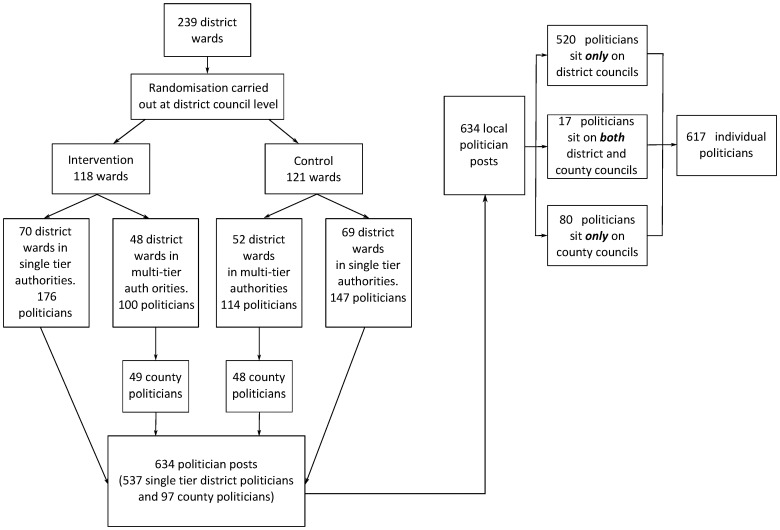
Distribution of local politicians (district and/or county) between intervention and control local authorities and electoral wards.

Figure 1 shows the distribution of the 617 politicians between the different tiers of local authorities included in the study.

**Figure 2:**
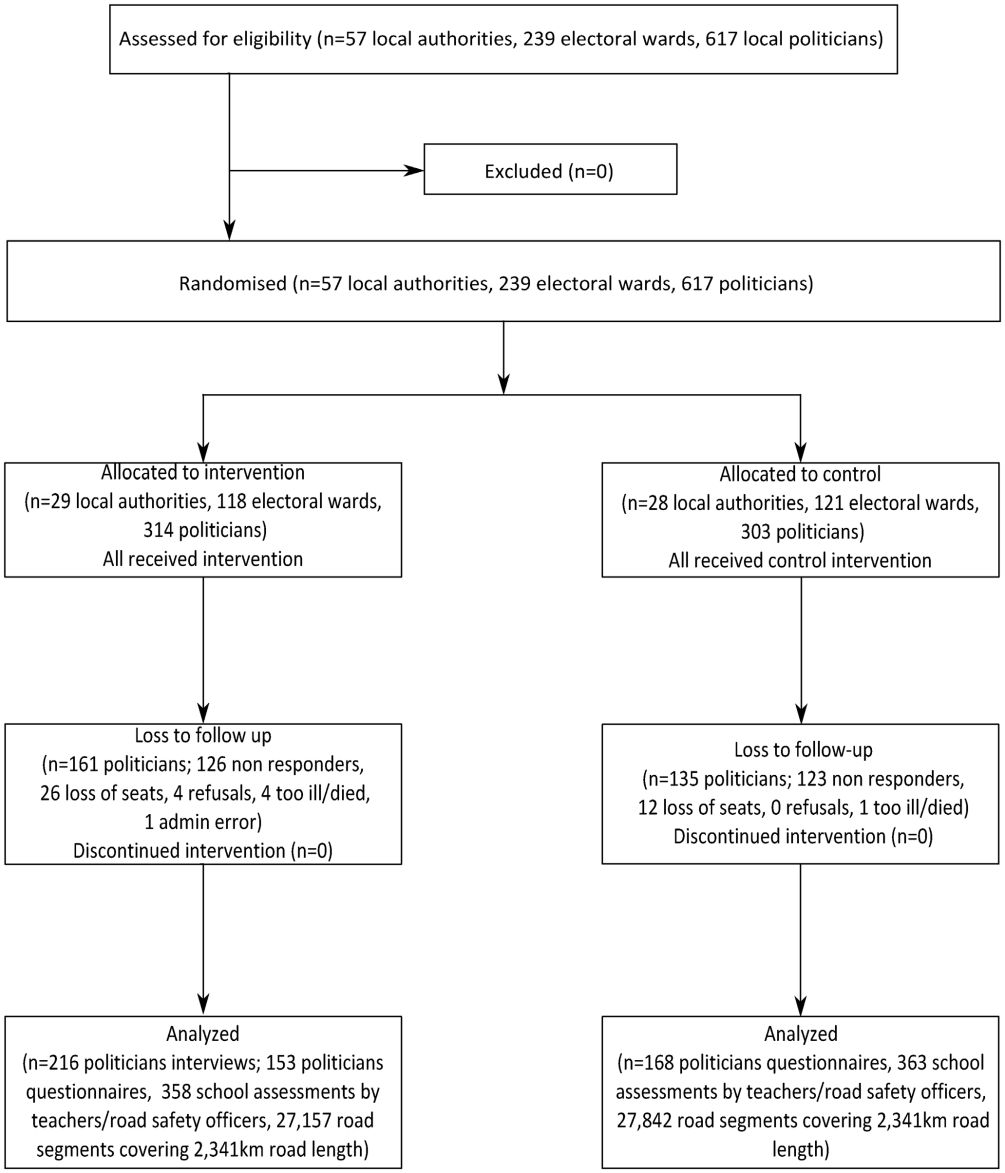
Flow of participants through the trial.

Figure 2 shows the distribution of local authorities, electoral wards and politicians in the intervention and control arms of the study. In total there were 617 politicians, representing 239 electoral wards in 57 local authorities. The mean number of wards per local authority was 4.2.

### Response Rates

Table 1 shows the numbers and response rates for all postal questionnaires and interviews with politicians. Response rates to postal questionnaires varied between 44–52% and between 59–69% for interviews.

**Table 1:** Response numbers and rates for all questionnaires and interviews with politicians.

Questionnaire/Interview	TotalSample	TotalContacted	% of totalsamplecontacted	Total responded	% of total contacted that responded	% of total sample that responded
1^st^ Postal Questionnaire (Control and intervention politicians)	617	617	100%	**273** (143 in C and 130 in I)	44.2% (47.19% in C and 41.40% in I)	44.2% (47.19% in C and 41.40% in I)
2^nd^ Postal Questionnaire (Control and intervention politicians)	617	569*	92.2%	**321** (168 in C and 153 in I)	56.4% (57.93% in C and 54.84% in I)	52.0% (55.45% in C and 48.73% in I)
1^st^ Telephone Interview (onlyintervention politicians)	314	314	100%	**216**	68.8%	68.8%
2^nd^ Telephone Interview (onlyintervention politicians)	314	310	98.7%	**185**	59.7%	58.9%

Councillors were not contacted for the following reasons; refusal to complete the 1^st^ questionnaire, loss of seat, illness and if the councillor was deceased.

Table 2 shows the numbers and response rates from the head teachers and road safety officers to the postal questionnaires. Responses were obtained from 73% of head teachers and 83% of road safety officers which provided information for 95% of schools.

**Table 2:** Response numbers and rates from head teachers and road safety officers to the postal questionnaires.

Questionnaire	Total Sample	Total responded	% of total sample that responded
Head teacher questionnaire	757	**553** (283 in C and 267 in I)	73.0% (72.94% in C and 72.36% in I)
Road safety officer questionnaire	757	**631** (300 in C and 331 in I)	83.4% (77.32% in C and 89.70% in I)
Questionnaire received from either the headteacher, road safety officer or both.	757	**721** (363 in C and 358 in I)	95% (93.56% in C and 97.02% in I)

### Baseline Data

Table 3 shows the baseline characteristics for intervention and control groups, illustrating that the groups appeared to be well balanced.

**Table 3:** Baseline characteristics of treatment groups. Values are numbers and % unless stated otherwise.

	Intervention Group n (%)	Control Group n (%)
**Local Authority level variables:**		
Number of local authorities	N = 29	N = 28
Study centre:		
South West	9	8
East Midlands	6	6
Surrey	10	9
South Wales	4	5
District council size:		
one to three wards	15	15
more than three wards	14	13
**Ward level variables:**		
Number of wards in group	n = 118	n = 121
The percentage of kilometres of road that are traffic calmed per ward (median, IQR)	3.2 (0.9 to 12.1)	3.2 (0.6 to 7.5)
**School level variables:**		
Number of head teachers in group	n = 369	n = 388
Number of responders	267 (72.4)	286 (73.7)
Number of district councils with responses	29	28
Number and percentage of schools with a Safe Route to School	14 (5.5) [10]	18 (6.5) [8]
Number and percentage of schools with a 20 mph zone	29 (10.9) [0]	23 (8.1) [1]

[] denotes missing values for responders to questionnaire.

### Main Results

Tables 4 and 5 show the results for the primary and secondary outcomes. There were no significant differences between the groups for the primary outcomes.

**Table 4:** Primary outcome measures by treatment group at 25–30 months post intervention.

Outcome	Intervention Groupn (%)	Control Group n (%)	Effect size (95% confidence interval){p value}
**Ward level variables:**			
Percentage of kilometres of road trafficcalmed per ward (median, IQR)	4.9 (1.8 to 13.9)	4.6 (1.1 to 8.6)	0.07 (−0.07 to 0.20)^1^ {0.32}
			Relative risk
Composite outcome measure of theproportion of wards where any new roadsafety interventions were introduced	104 (100.0) [14]	108 (100.0) [13]	Cannot be estimated
**School level variables:**			
Number (%) of schools having 20 mph zones	98 (27.7) [3]	66 (18.8) [12]	1.47 (0.93 to 2.32) {0.10}
Number (%) of schools having a Safe Routeto School initiatives	71 (20.2) [5]	52 (14.4) [4]	1.34 (0.83 to 2.17) {0.23}
Number (%) of schools providing practical pedestrian training	111 (31.3) [2]	114 (31.3) [0]	1.23 (0.95 to 1.61) {0.12}
Number (%) of schools providing other road safety education	229 (65.6) [8]	210 (59.8) [13]	1.16 (0.97 to 1.39) {0.09}
**Local politician level variables:**			
Number (%) of local politicians who have lobbied for physical road safety measures or more road safety education in their wards.	133 (86.9) [0]	142 (84.5) [0]	1.02 (0.94 to 1.11) {0.63}

[] denotes missing values for responders to questionnaire.

1 regression coefficient, using cube root transformation.

**Table 5:** Secondary outcome measures by treatment group at 25–30 months post intervention.

	Intervention Groupn (%)	Control Groupn (%)	Relative risk (95% confidence interval) {p}
**School level**			
Number (%) of schools at follow-up with 20 mphzones planned	10 (3.8) [0]	20 (7.0) [1]	0.52 (0.23 to 1.16)† {0.11}
Number (%) of schools at follow-up planning a Safe Routesto School initiative	38 (14.5) [4]	37 (12.9) [0]	1.26 (0.84 to 1.89)† {0.27}
Number (%) of schools at follow-up planning to providepractical pedestrian training	11 (4.1) [1]	15 (5.2) [0]	0.84 (0.42 to 1.68)† {0.62}
Number (%) of schools at follow-up in process of making aschool travel plan	43 (16.1) [0]	46 (16.1) [0]	0.98 (0.63 to 1.52)† {0.92}
Number (%) of schools at follow-up planning one or more of measures above	76 (28.9) [0]	89 (31.2) [1]	0.94 (0.70 to 1.26)† {0.67}
**Local politician level**			
Interested in child injury prevention	123 (94.6)	122 (85.9) [1]	1.09 (1.03, 1.16)* {0.003}
Involved in child injury prevention in the last 12 months	49 (38.0) [1]	35 (25.0) [3]	1.50 (1.08, 2.09)* {0.02}
Believes could take action to help prevent child injuries intheir ward	94 (73.4) [2]	76 (53.5) [1]	1.36 (1.16, 1.61)† {<0.001}
Mentioned pedestrian safety as one action for preventingchild injuries in their ward	62 (53.0) [13]	45 (33.3) [8]	1.55 (1.19, 2.03)† {0.001}

[] denotes missing values for responders to questionnaire.

Coding of interest in child accident prevention: Yes = Very interested/interested, No = Neither interested or uninterested/not interested/not at all interested.

* estimated using Poisson generalised estimating equations.

† estimated using log-binomial generalised estimating equations.

Among the secondary outcomes politicians in the intervention group reported increased interest (RR 1.09; 95%CI 1.03 to 1.16), greater belief that they could take action to reduce child injuries in their ward (RR 1.36; 95% CI 1.16 to 1.61), more involvement in injury prevention (RR 1.50; 95% CI 1.08 to 2.09) and greater identification of pedestrian safety interventions suitable for their areas (RR 1.55; 95%CI 1.19 to 2.03).

### Ancillary Analyses

Ancillary analyses of survey data were undertaken to provide contextual information. In the postal survey undertaken 1–3 months following commencement of the intervention, local politicians were asked about twenty three issues in their wards (Table 6). Speeding was the 2^nd^ most commonly mentioned problem, reported by 78% of politicians. When interviewed at 1–3 months following commencement of the intervention 68% (147) of intervention group politicians reported that the information in the packs was ‘surprising’ and 65% (138) reported that they were either ‘fascinated’, ‘interested’ or ‘very interested’ in the information. Sixty percent (150) agreed that the information pack was ‘effective’ or ‘very effective’ in presenting road safety information whilst 9% (19) felt that it was either ‘ineffective’ or ‘very ineffective’. Nearly half (48%, n = 104) wanted more information with many wanting more detailed maps or times and dates of incidents, with some (12%, n = 25) calling for the publication of national league tables. Most local politicians (77%, n = 163) reported that they could identify interventions suitable for their wards.

**Table 6:** Issues reported as being a ‘very big’ or ‘fairly big’ problem in their wards by local politicians at 1–3 months after baseline.

Question asked: “To what extent do you consider the following to be problemswithin your ward?” Issues are ranked by frequency.	Number (%) of politicians that considered the following factors to be a problem in their ward.
Teenagers hanging around on the streets	209 (78.0) [5]
Speeding motorists	209 (77.7) [4]
People using or dealing drugs	198 (75.0) [9]
Vandalism, graffiti and other deliberate damage to property	191 (70.0) [0]
Rubbish or litter lying around on the streets	171 (63.6) [4]
People being drunk or rowdy in public places	153 (58.0) [7]
Insufficient leisure facilities	148 (56.7) [12]
Unemployment	129 (48.9) [9]
Inadequate maintenance of paths	113 (43.8) [15]
Insufficient safe playgrounds	111 (43.0) [15]
Inadequate public transport	100 (37.9) [9]
Car theft	100 (37.6) [7]
Domestic violence	94 (37.2) [20]
Homelessness	98 (37.1) [9]
Burglaries	96 (36.4) [9]
Noisy neighbours or loud parties	94 (35.3) [7]
Joy riding	91 (35.0) [13]
Road accidents	87 (33.2) [11]
Poor quality housing	86 (33.1) [13]
Abandoned and burnt out cars	66 (24.9) [8]
Assault/mugging	52 (19.7) [9]
Accidental injuries in the home	24 (10.1) [35]
House fires	11 (4.4) [24]

[] denotes missing values for responders to questionnaire.

Coding of problems in ward: Yes = Very big problem/Fairly big problem, No = Not a very big problem/Not a problem at all.

At 17–22 months following commencement of the intervention 63% (n = 117) of intervention group politicians reported being involved in lobbying or supporting pedestrian safety schemes in their areas. Three quarters identified specific barriers to improving safety, principally funding (40%, n = 75) and some mentioned lack of political will (9%, n = 16), problems with council structures (6%, n = 11) and occasionally unsupportive attitudes of officials (10%, n = 18).

## Discussion

### Principal Findings

This study has shown that a targeted approach to engaging elected local politicians, representing deprived communities with high pedestrian injury rates, is effective in increasing their interest and involvement in advocating for improved safety measures in local areas. However, this did not lead to a significantly increased implementation of road safety measures over a 25–30 month period. The findings of this study provide evidence that local politicians recognise that road safety and speeding are major concerns in deprived communities. They are receptive to information about risk in their areas and the majority report being willing to advocate for improved safety interventions.

### Strengths and Weaknesses of the Study

The Advocacy for Pedestrian Safety Study represents a rigorously designed and implemented cluster randomised trial. The intervention was based on sound theoretical individual-level behaviour change models and the acceptability of the messages was successfully piloted with politicians from other areas prior to adoption. [23] Primary outcomes were collected in an unbiased manner as data were either obtained from independent sources (traffic calming) or from road safety officers and teachers blinded to intervention status.

Cost restrictions on the design of the study meant that information on secondary outcomes collected by semi-structured interviews 17–22 months after the intervention could only be collected from intervention politicians and it was not possible to determine what proportion of control politicians would also have reported being involved in supporting safety interventions. We explored the potential for verifying self reported involvement in road safety interventions through the use of council minutes and websites, but these varied greatly across councils and hence were not considered sufficiently reliable for use.

Another important limitation of this study was the length of time it was possible to follow up outcomes. The four year grant which supported this work meant that it was possible to follow up the primary outcome (traffic calming) only to 25–30 months post intervention. Given the time it takes to design the intervention and to affect change through council planning structures our study may have been too short to detect important effects. Longer term research funding streams are required to evaluate complex interventions with long time frames.

### Strengths and Weaknesses in Relation to Other Studies, Discussing Important Differences in Results

This study represents an innovative approach and a rare example of translational public health research using political advocacy as a tool to improve the uptake of effective interventions for high risk groups in deprived communities. [20] There appears to be only one previously published paper of an evaluation of a political advocacy approach to improving child health tested within a randomised trial in Illinois, US. [31] Whilst there are some similarities between this study and ours, there are also important differences. The Illinois study involved sending a letter to senators prior to a vote on child safety restraints in 1982. In that study, 79% of 29 senators in the intervention group voted for the bill, compared to 53% of the 30 senators in the control group (p<0.05). [31] The results of the secondary outcomes of our study are consistent with this finding, with those in the intervention group reporting significantly greater interest in child injury prevention, and belief that they could take action to improve pedestrian safety in their localities. The positive impact on senator activity in the Illinois trial may differ from our findings for our primary outcome measures as the Illinois trial required only a single action to be undertaken shortly after the delivery of the intervention. Demonstrating changes to road safety infrastructure in our trial would have required repeated local politician activity over a long period of time, the commitment of finances and the planning and provision of infrastructure changes, which is likely to be much more difficult to achieve. We believe our study is unique in randomly allocating elected local politicians to intervention and control groups and attempting to influence non legislative activities to improve public health through the implementation of effective interventions.

### Meaning of the Study: Possible Explanations and Implications for Clinicians and Policy Makers

Skills in political advocacy are needed by clinicians and policy makers in implementing evidence based practice, particularly in resource constrained times. That the public health function in England is moving from the NHS to local authorities further emphasizes the importance of political advocacy skills for public health practitioners. [41] These groups can learn much from the Advocacy for Pedestrian Safety Study which successfully engaged with local politicians and resulted in increased support for improving safety. However, as it did not change road safety measures within the trial time frame, the reasons behind this limited effectiveness need to be understood to inform the development of further approaches promoting advocacy or to consider other approaches to improve pedestrian safety in high risk deprived communities.

The advocacy package proved to be acceptable and interesting to local politicians. Most were surprised by the high casualty rates in their wards, suggesting a lack of awareness of the magnitude of road traffic risks in their localities. This is not surprising as such maps and analyses have not been previously shown to politicians, and is consistent with our findings that road traffic injuries were reported as a problem in their ward by only 33% of local politicians. Interestingly, speeding motorists were reported as a problem by 78% of local politicians, suggesting some degree of disconnect between their understandings of the two issues. The majority of politicians thought the advocacy pack was effective in presenting road safety information, but many also requested more detailed information. The pack also appeared to stimulate identification of interventions that would be suitable for their wards and subsequent action, with 63% reporting lobbying or supporting pedestrian safety schemes. Whilst these results are encouraging they are from unverified self reports and could be susceptible to reporting bias.

There are of course many barriers to the introduction of pedestrian safety schemes, many of which were recognised by local politicians. These include lack of available finance, competing priorities, long delays in planning or implementing schemes through complex council structures, diffuse representative structures, and sometimes lack of supportive attitudes from officials who are often under pressure from inadequate resources and competing demands. Within councils, many people are involved in decision making. Responsibility of road safety might sometimes be perceived to be the domain of largely unelected safety partnerships external to representative structures. Power to influence change may be located within different individuals or departments, and not necessarily in departments dealing with road safety. Previous research showed that influence is unequally distributed between local politicians, with more traffic calming than expected in areas represented by politicians occupying key decision making posts. [22] Multi-level local government structures are a further complication in some areas. Responsibility for road safety may be held at different tiers of local government, limiting the potential for politicians representing one tier to influence the actions of other tiers.

The UK Audit Commission’s ‘Changing Lanes’ report suggests that there is a prevalent view in road safety departments that returns from road safety engineering are diminishing because the main black spots and dangerous stretches of road have already been treated by traffic calming. [42] Recent research however found only 3.7% of road surface is traffic calmed suggesting considerable potential for further engineering approaches to speed reduction. [16] Despite this, it is possible that perceptions about diminishing returns from road safety engineering may have influenced road safety and engineering departments limiting local politicians’ ability to affect road safety interventions in their wards. Due to relatively long planning cycles it is also likely that the implementation of previously planned interventions in intervention and control wards will have limited the ability of this study to demonstrate the effectiveness of the advocacy approach. Despite strenuous efforts, we found it impossible to find detailed information on what interventions were planned; variability in council structures, responsibilities and lack of standardised record keeping contributed to this situation.

Our study demonstrates that road safety provision changed considerably in deprived wards in England and Wales between 2005 and 2008, starting from a very low base. A 50% increase in the median proportion of road length traffic calmed took place; the provision of 20 mph zones around schools also increased by 50% and the numbers of Safe Routes to Schools tripled. However, despite these increases, by the end of the study less than 5% of all roads in wards with high pedestrian casualty rates were traffic calmed, more than three quarters of local authorities still had no provision for 20 mph zones around schools and only 17% of schools had Safe Routes to School. This clearly demonstrates that the provision of effective road safety interventions is still inadequate, even in those areas of greatest need.

It is difficult to know whether local politicians circulated the information and advocacy packs widely within council planning structures or elsewhere. Certainly, in a number of locations the information found its way to the local media which helped to highlight the issues. A sizeable minority of local politicians requested more detailed and up to date information and maps and some (12%) called for the publication of national league tables. Placing such information in the public domain would also allow other groups to advocate for action and could be particularly helpful when the discrepancy between injury risk and safety investment is large.

### Generalisability

The overall design and methodology used in this study should be of interest to clinicians, policy makers and public health advocates in many settings. We have demonstrated that it is possible to design and implement a cluster randomised trial of political advocacy. The factors influencing local politicians’ interest in, and behaviour towards, road safety and the barriers which they face in effectively advocating for safety interventions will be relevant in many jurisdictions across the world. Inequality in road traffic injury is a global issue. [3] The specific findings of this study should also be generalisable to other areas of the UK and to countries with similar political structures and resources. The study sites were chosen to be within 50Km of several research centres and covered a wide area; the patterns of road collisions and safety interventions are likely to be similar across the UK.

### Unanswered Questions and Future Research

There are many barriers to implementing pedestrian safety measures, including a dearth of effective local advocacy groups, perhaps due to the absence of publically available information on the scale of injuries and preventive interventions at local levels. Were such information to be made available it is likely that communities at high risk of injury but with few or no protective interventions would be much more effective in lobbying for change. Building on this study’s findings, the Injury Observatory for Britain and Ireland has proposed that such information should be routinely available to the general public through the development of a ‘SafeArea’ website. [43] This initiative which is being developed in a pilot site may provide the basis for the development and evaluation of modified approaches to public health advocacy.

This study has shown that the design and implementation of an advocacy package on road safety is feasible within the context of the UK. Further research needs to focus on how advocacy packages can be adapted to generate more action from local politicians; for example, how local media and local community advocates could be involved and whether greater reinforcement of the messages of the package is needed. Case studies using qualitative methodologies documenting the process by which successful implementation of safety measures are carried out would be helpful in informing the design of further intervention trials. Future studies should be carried out over much longer follow-up periods to allow for inevitable delays inherent in planning and delivering safety interventions, particularly those requiring engineering work. Longer-term studies would also facilitate the use of qualitative methodologies at an intermediate stage which could be used to decide whether there was a need to refine intervention strategies mid trial.

## Supporting Information

Appendix S1 An example of an intervention package.
(TIFF)

Appendix S2 Control group information package.
(TIFF)

Protocol S1 Published study protocol.
(PDF)

Checklist S1 Supporting CONSORT checklist.
(DOC)
