# Supplementary figures and images for: The Advocacy for Pedestrian Safety Study: Cluster Randomised Trial Evaluating a Political Advocacy Approach to Reduce Pedestrian Injuries in Deprived Communities
Source: PLoS One. 2013 Apr 8;8(4):e60158. doi: 10.1371/journal.pone.0060158 (PMC3620122; doi:10.1371/journal.pone.0060158)

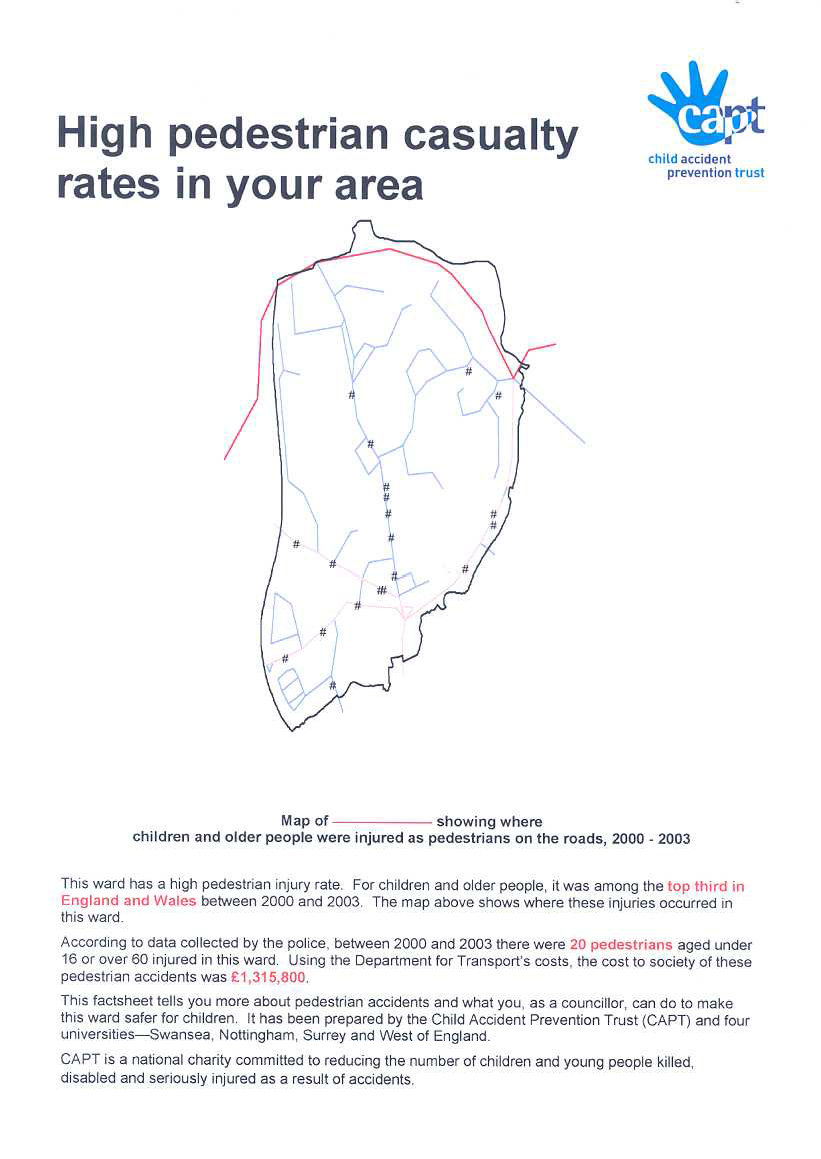

Supplement: Appendix S1 — An example of an intervention package. (TIFF) [file pone.0060158.s001.tiff]

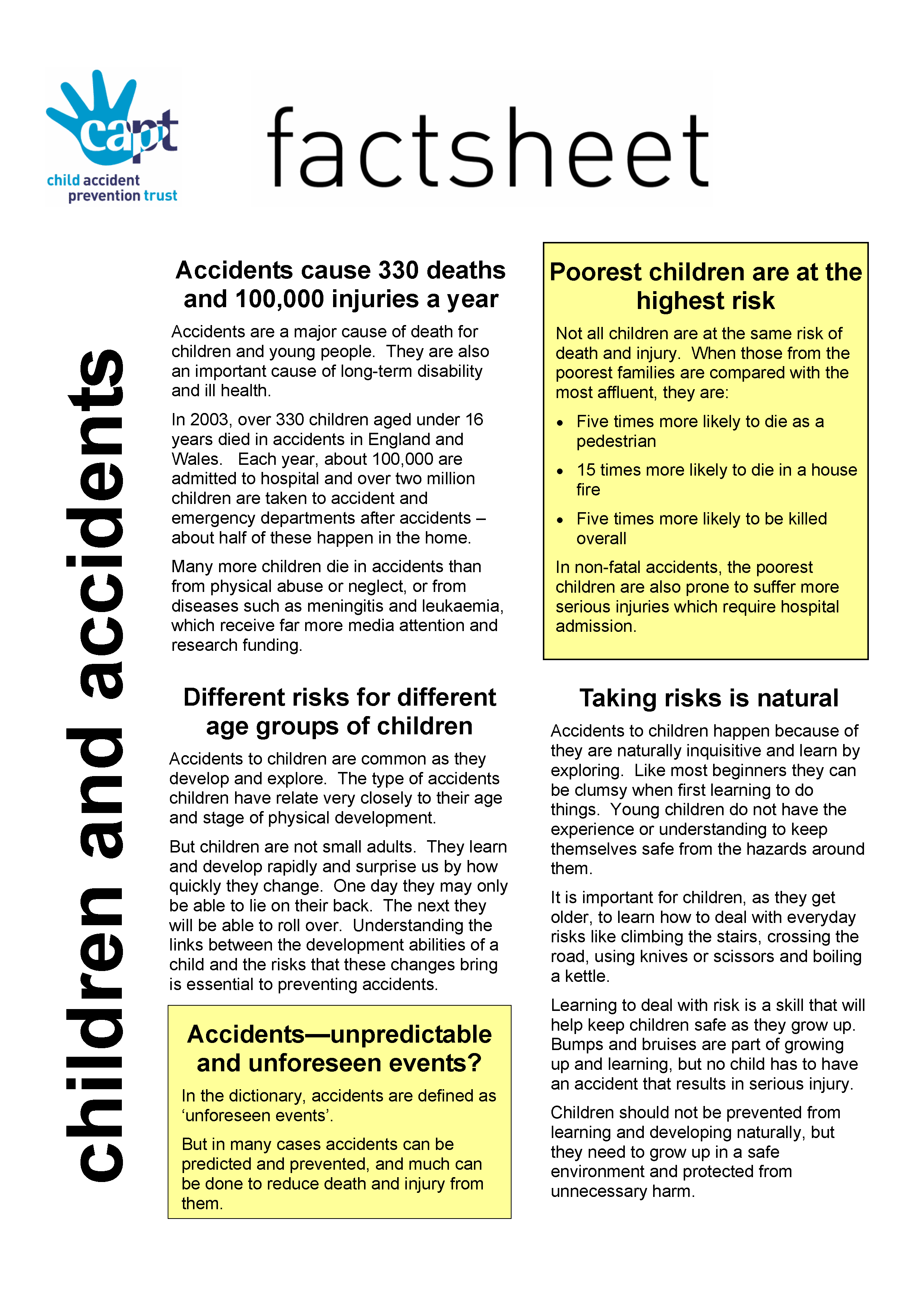

Supplement: Appendix S2 — Control group information package. (TIFF) [file pone.0060158.s002.tiff]
