# Supplementary material for: The Advocacy for Pedestrian Safety Study: Cluster Randomised Trial Evaluating a Political Advocacy Approach to Reduce Pedestrian Injuries in Deprived Communities
Source: PLoS One. 2013 Apr 8;8(4):e60158. doi: 10.1371/journal.pone.0060158 (PMC3620122; doi:10.1371/journal.pone.0060158)
Supplement: Protocol S1 — Published study protocol. (PDF) [file pone.0060158.s003.pdf]

MIXED METHODS STUDY INCORPORATING A RANDOMISED TRIAL OF THE USE OF INFORMATION AND ADVOCACY WITH LOCAL COUNCILLORS AND LOCAL DIRECTORS OF PUBLIC HEALTH TO REDUCE INEQUALITIES IN INJURY RISK.

STUDY PROTOCOL

Background

Few interventions have specifically been used to target injury inequalities and the overall success of different interventions has been variable. Logically, traffic calming, specifically positive physical controls that force drivers to slow down or change direction, should reduce injury rates and, if located primarily in areas of greatest need, should reduce inequalities in injury rates. Although few studies exist measuring its effectiveness, indications are that area wide traffic calming may be a promising intervention to reduce overall road traffic deaths and injuries[1]. With government policies focussing increasingly on reduction in health inequalities it is appropriate to look at the social distribution of safety measures and to see whether traffic calming policies might influence inequalities in childhood pedestrian injuries.

In a study, targeted traffic calming in the deprived areas of one city were associated with a substantial reduction in pedestrian injury inequalities compared with a control city where interventions were more evenly distributed[2]. Analysis showed that in both cities there was substantially more traffic calming in the home area of influential councillors. This finding shows the importance of local decision making and the necessity to engage at this level to reduce inequalities in injury occurrence. No other examples of evaluated interventions exist in the injury field which have focussed on using an advocacy package targeted at local decision makers.

This proposal includes inequality observation, intervention development and testing. Our aim is to quantify and reduce inequalities in road traffic accidents among road users in four intervention areas and thus providing greater insights into local decision making processes. The framework will be developed and tested, in the field of pedestrian safety. We propose to focus on how best to bridge the inequalities gap by providing more informed and more targeted traffic calming measures and other safety interventions to those wards with the greatest pedestrian injury rates. This involves calculation of injury rates in small areas and provision of information on the size of the local problem, together with targeted advocacy by the Child Accident Prevention Trust (CAPT) on effective interventions aimed at local Directors of Public Health and elected representatives (local councillors). This framework could potentially be extended across all types of injuries and to health promotion generally.

Objectives

- To study injury rates and inequalities in road traffic accidents involving vulnerable road users at regional and local levels.
- To map the road traffic environment (i.e. the location of existing road traffic calming facilities) in the four intervention areas.
- To document the reporting of road traffic accidents and of traffic calming measures in the four areas preceding the implementation of the intervention.
- To develop an advocacy package comprising a core package for all 4 areas and tailored information containing local information for the 4 sites.
- To carry out a mixed method study of advocacy, encouraging Directors of Public Health and local community leaders to improve safety of the road traffic environment in their areas in the context of a randomised controlled trial.

Methods

Setting

The intervention will be a randomised controlled trial design and implemented in three regions of England (South West, East Midlands, South East) and in parts of Wales.

Study population

Local public health directors and local councillors of wards within the intervention areas where the local authorities have 40% or more wards in the most deprived fifth.

Inclusion and exclusion criteria

The following participants will be included:

- Local public health directors within the study population.
- Local councillors of wards within the study population

The following participants will be excluded:

- Local public health directors not within the study population
- Local councillors of wards not within the study population

Sample Sizes

The numbers of deprived wards in the four regions are:
East Midlands region, 7 LAs with 40%+ wards most deprived, 95 most deprived wards
South West, 4 LAs with 40%+ wards most deprived, 62 most deprived wards
South East, 2 LAs with 40%+ wards most deprived, 24 most deprived wards
Wales, 5 LAs with 40%+ wards most deprived, 78 most deprived wards.

Pilot areas

A pilot study has been carried out in two areas of Wales which measured the frequency of traffic calming (TC) features in Wards and found that there were on average 21 features per Ward. A standardised difference of 0.35 between the mean number of new interventions in intervention and control areas would be of public health importance. Using this as an important measure of effect then a 1 sided significance test (reasonable assumption that interventions can only improve uptake) at $\alpha = 0.05$ and power of 80% requires 102 Wards in each arm of the trial. The wards are however clustered within local authorities. To achieve this number an average of 5 wards can be selected from 20 local authorities giving a cluster size of 4. The intra class correlation coefficient is not yet known but similar analysis at postcode level in the Health Survey for England suggests that an ICC of 0.05 would be conservative. This yields a design effect of 1.15 and a sample size of 117[3]. Rounding up to 120 would therefore retain the same power. This number is achievable, given the numbers above, but a larger number of intervention sites might not be logistically possible within the tight financial limits.

Groups will therefore comprise of 30 control and 30 intervention wards in each region - 120 wards in total on each arm. Randomisation will be at the local authority (LA) level and using high injury rate ward data and maps.

Data and data analysis

Police road traffic casualty data (STATS 19) for 2000, 2001, 2002 and 2003 will be combined and analysed at national, regional and local levels. Analysis ward level will use denominator data from the 2001 census. Ward based deprivation profiles generated from the Townsend Index of Material Deprivation, Index of Multiple Deprivation (IMD) and Welsh IMD and ethnicity profiles generated by the 2001 census will be used.

Study outline and timetable

January 2005 – June 2005:
To assist in the development of an advocacy package and questionnaires, focus group meetings comprising of local councillors, (drawn from two local councils in areas geographically separate from the proposed trial areas) will be conducted. Issues relating to traffic calming and pedestrian injuries in vulnerable groups will be discussed, reports from local newspapers will be utilised in this process. In addition to a meeting facilitator, an observer will document the proceedings and summarise the main findings. This information will be used in the development of an information pack for councillors. The information pack will be piloted with members of the focus group. We will also use external advisors to help inform this process who have considerable experience of local authority work e.g. Professor Jo Sibert, previous mayor and long term councillor in Penarth, and Mr Andrew Jones, DPH in Denbighshire with long term experience in environmental health with various councils.

May/ June/ July 2005:

i. Injury rate profiles by deprivation fifth at the regional and local authority levels will be generated by analysing STATS19 data. The 120 highest pedestrian injury wards in deprived areas will be identified.
ii. The road traffic environment of 60 wards in each region (240 wards in total) will be mapped, initially for a baseline survey. Accident locations and features e.g. traffic calming, school crossing patrols, safe routes to school, road bendiness, playground locations will be mapped and distribution between high and low injury rate areas compared to identify possible reasons for rate variations.

September 2005:
Following an initial telephone call outlining the study in order to make contact, a baseline questionnaire will be issued to local councillors and directors of public health (DsPH) within the relevant primary care trust (PCT) and a follow–up email (after 2 weeks) and a telephone(after 3 weeks) contact for non-responders.

October 2005:
The ***intervention group*** will receive an information pack sent from CAPT. The pack (subject to focus group work and piloting) will contain information concerning: national and local road accidents, inequalities of pedestrian injuries and the population at risk, pedestrian injury risk factors, evidenced based preventative measures, the problem of obesity and the need for walking in safe environments, government policies e.g. 'Tomorrow's Road Safer for Everyone', media cuttings, the potential role of the council, case histories, cost of injuries and cost benefits of injury prevention and local Road Safety Officer contact details. The intention is that the pack will encourage action by these individuals and using evidence generated from maps participants will be able to identify where traffic calming measures are concentrated within their area.

The ***control group*** will receive a fact sheet sent from CAPT. The fact sheet will contain general information concerning: children's accidents, inequalities of injuries, preventative measures and government policies.

November 2005 – January 2006:
The telephone interviews will be conducted with intervention group participants in relation to the information pack – how they found it, was the information helpful or not and to reinforce the safety message.

March 2006:
A follow-up postal questionnaire will be issued to all participants to determine if any changes have occurred.

March 2006, March 2007, March 2008

Minutes of council meetings in the four areas will be sort after trawled for mention of 'road safety' and other associated terms.

The road safety officer in each LA will be contacted in relation to safe routes to school, kerbcraft etc.

February / March 2007:

A telephone interview will be conducted with intervention group participants to obtain a longer term view of whether the intervention helped of hindered and identify barriers and successes.

October/November 2007:

Changes in the physical road traffic environment will be assessed by repeating the mapping exercise in the 240 wards for post intervention comparisons.

December 2007 – March 2008:

Data from the interviews, questionnaires and mapping exercises will be analysed and the final report will be written up at the study office.

Main outcome measures

- The adoption or initiation of safety schemes e.g. traffic calming, speed cameras, safe routes to school, new educational interventions in intervention and control areas at 31 months.
- Changes in attitudes to road safety.

Dissemination

All results will be submitted to peer reviewed journals, CAPT and the Department of Health.

References

1. Bunn, F., Collier, T., Frost, C., Kerr, K., Roberts I. and Wentz R. Area wide traffic calming for preventing traffic related injuries (Cochrane Review). In *The Cochrane Library*. Issue 1, 2003. Oxford.
2. Jones, S.J., Lyons, R.A., John, A., Newcombe, R. and Palmer, S.R. Child pedestrian injuries – reducing inequalities using traffic calming. Injury Prevention 2005 (In press).
3. Gulliford M.C., Ukoumunne O.C., Chinn S.
   Components of variance and intraclass correlations for the design of community-based surveys and intervention studies. Am *J Epidemiol* 1999;149:876-83.
